# Evaluation of the Antidepressant Effect of the Functional Beverage Containing Active Peptides, Menthol and Eleutheroside and Investigation of Its Mechanism of Action in Mice

**DOI:** 10.17113/ftb.58.03.20.6568

**Published:** 2020-09

**Authors:** Yuanjin Qi, Huizhen Zhang, Sha Liang, Jiajia Chen, Xiaoni Yan, Zhouyu Duan, Deyang Zhou, Zhicheng Li

**Affiliations:** 1 College of Food Science and Engineering, Northwest A&F University, 22 Xinong Road, 712100 Yangling, PR China; 2 Department of Food Science, University of Tennessee, 2510 River Dr, TN 37996 Knoxville, USA

**Keywords:** functional beverage, eleutheroside, behavioral testing, antidepressant mechanism, monoamine, antioxidation

## Abstract

**Research background:**

Depression has become a global threat to human health. In order to solve it, researchers have conducted multi-faceted studies including diet. Many food-derived bioactive substances have shown antidepressant effects. However, there are few studies on the design of industrialized food with antidepressant effect. This study aims to evaluate the antidepressant effect of a functional beverage made from several ingredients with potential antidepressant function and investigate its antidepressant mechanisms.

**Experimental approach:**

The beverage consists of peppermint oil, active peptides derived from bovine milk casein and *Acanthopanax senticosus* extract (ASE) whose active ingredient is eleutheroside. Different amounts of ASE were evaluated to determine the optimal concentration of eleutheroside in this functional beverage to deliver the best antidepressant effect through extensive behavioral testing, including preliminary acute stress experiments and further chronic unpredictable mild stress test.

**Results and conclusions:**

The results demonstrated that the beverage with 15 mg/kg of eleutheroside could significantly reduce the mice’s immobility time of tail suspension test and forced swimming test, recover mice’s sucrose preference and behavior changes in the open field test, improve the contents of dopamine, norepinephrine, 5-hydroxytryptamine and the activity of superoxide dismutase and reduce the content of malondialdehyde in mice’s brains, which indicated that the improvement of monoamine neurotransmitter systems and antioxidation was one potential mechanism of antidepressant action.

**Novelty and scientific contribution:**

This study provides a design of antidepressant functional beverage and an efficient way for the prevention and treatment of depression.

## INTRODUCTION

Major depressive disorder (MDD) is a serious mood disorder, which can be caused by a combination of biological, psychological and social distress. Patients suffering from depression often have unstable emotions, with long-lasting symptoms that usually deprive the patient’s capabilities for work and logical communication (*1*). Depression could even lead to suicide. According to the World Health Organization (WHO), 850 000 people commit suicide every year due to depression (*2*). The WHO had predicted that depression would be one of the two top causes of global health disorders and disability (*3*).

In addition to common nutrients, foods are also sources of bioactive substances that have a potential positive impact on human health (*4*). Diet may provide considerable benefits for moderate to severe depression and anxiety (*5*). According to reports, some Chinese herbal medicines and fruits also have certain antidepressant effects (*6*-*8*). Mint, the dried aerial part of the Lamiaceae plant *Mentha haplocalyx* Briq., is cool-natured, with acrid flavour, and it can disperse stagnated liver in the beliefs of Traditional Chinese Medicine (*9*). L-menthol is the main active ingredient of the mint, and its content is up to 87% in the essential oil of mint (*10*). It was able to induce an antidepressant-like effect in a mouse model of depressive behavior, and this effect might be partially mediated by dopaminergic (DAergic), 5-hydroxytryptaminergic and gamma-aminobutyric acidergic pathways (*9*). *Acanthopanax senticosus* Harms (ASH) root bark is also traditionally used to treat high blood pressure and mental disorders in China (*11*, *12*). ASH has an anxiolytic effect against not only mild anxiety, but also anxiety due to higher levels of stress, which is related to an increase in hippocampal brain-derived neurotrophic factor signaling (*13*). Bioactive peptides are small protein fragments derived from enzymatic hydrolysis and gastrointestinal digestion of food proteins, which are beneficial to living beings (*14*). Among the many protein foods, milk is a major research object (*15*). Kim *et al.* (*16*) have shown that the ingestion of alpha(S_1_)-casein hydrolysate can decrease the stress-related symptoms in females, particularly in intellectual and emotional problems. Guesdon *et al.* (*17*) demonstrated that in mice the tryptic bovine alpha(S_1_)-casein hydrolysate has protective effect on sleep during exposure to chronic mild stress conditions. In recent years, the demand for foods with additional functional benefits has been increasing (*18*). However, the design of industrialized food with antidepressant effect has been rarely explored.

In the present study, a beverage (referred as functional beverage below) was designed, which consisted of peppermint oil, active peptides derived from bovine milk casein and *Acanthopanax senticosus* extract (ASE) whose active ingredient is eleutheroside. In order to fully confirm antidepressant effect of the functional beverage in mice and determine the optimal concentration of eleutheroside in the functional beverage to deliver the best antidepressant effect, we conducted extensive behavioral testing including preliminary acute stress experiments and further chronic unpredictable mild stress (CUMS) test. The potential antidepressant mechanisms were also investigated.

## MATERIALS AND METHODS

### Preparation of functional beverage

We chose bovine milk casein hydrolysates, peppermint oil (Jinxing Spice, Dongtai, Jiangsu, PR China) and *Acanthopanax senticosus* extract (ASE; Hongda Plant Chemical, Xi’an, Shaanxi, PR China) as the ingredients of functional beverage and their concentration settings referred to some reports and a Chinese national food standard (*19*-*21*). The functional beverage was made of peppermint oil (0.30 g/kg), bovine milk casein hydrolysates (690 g/kg) and ASE (0.25-2.00 g/kg), which contained, respectively, 0.10 g/kg menthol, 25.05 g/kg active peptides and 3.75-30.00 mg/kg eleutheroside, consisting of equal amounts of eleutheroside B and eleutheroside E.

We prepared and determined bovine milk casein hydrolysates in our previous study with the same enzymes and reagents (*22*). The protein content of bovine milk casein hydrolysates was determined to be 4.06% (by mass) by Kjeldahl method (*23*). The trichloroacetic acid precipitation method (*24*) was used to measure the peptide mass fraction of the hydrolysates, which was 3.63% (by mass). The hydroxyl radical-scavenging activity of bovine milk casein hydrolysates was determined to be 50.06% by using the 2-deoxy-d-ribose oxidation method (*22*). The mass fractions of menthol in peppermint oil and eleutheroside in ASE were respectively 33.33 and 1.50%, which were obtained from the guaranteed analysis provided by the manufacturer. In the following text, all mass fractions refer to the mass fraction of eleutheroside in the functional beverage.

### Animals

Male Kunming mice (20-25 g) and fodder were provided by the experimental animal center of Fourth Military Medical University (Xi’an, Shaanxi, PR China). The production license number of experimental animal was SCXK (Shaanxi) 2014-002. The fodder was standard mice pellet feed. The rearing environment was a specified pathogen-free laboratory animal room with the license number of experimental animal SYXK (Shaanxi) 2014-001. All animals were housed under standard conditions of temperature (22±2) °C, humidity (55±4) % and light (12:12 h light/dark cycle), and free access to food and water. Clomipramine hydrochloride (CH; Weimeng Biotech, Shanghai, PR China) was used in the present study. All animal use procedures were carried out in accordance with the Regulations of Experimental Animal Administration issued by the State Committee of Science and Technology of the People’s Republic of China (*25*), with the approval of the Northwest A&F University Ethical Committee. All behavioral experiments were performed once.

### Preliminary acute stress experiment

#### Treatments in acute stress experiment

Acute stress experiment in the present study included forced swimming test (FST) and tail suspension test (TST), which are used widely to measure the pharmacological effects of antidepressant drugs or changes in stress-evoked behavior in mice (*26*, *27*). They were carried out in order to determine the preliminary mass fraction with a better antidepressant effect. In each test, fifty experimental mice were randomly divided into 5 groups (*N*=10). They were fed with normal saline (control group), 40 mg/kg CH (CH group), and three functional beverage samples (7.50, 15.00 and 30.00 mg/kg of eleutheroside). All animals in each group were fed twice a day with intragastric administration of 0.02 mL/g body mass every time for 5 days continuously. The mice were not anesthetized before gavage. Mice that were subjected to TST and FST were sacrificed by cervical dislocation.

#### Tail suspension test

The TST was conducted 1 h after the last intragastric administration on day 5. Mice were suspended for 6 min by placing an adhesive tape 1 cm away from the tip of the tail. Each mouse was suspended 50 cm away from the floor and was acoustically and visually isolated from other animals during the test. The immobility time of each mouse was subsequently recorded. Immobility was defined as when the mouse’s four paws and head were all immobile or passively swinging (*28*).

#### Forced swimming test

The FST was conducted 1 h after the last intragastric administration on day 5. Each mouse was placed into an 80-litre polypropylene basin (height 24 cm, diameter 65 cm) filled with 50 L of water at (25±1) °C. The mouse was forced to swim for 6 min and judged to be immobile when it floated in an upright position and made only small movements to keep its head above water. The duration of immobility was recorded during the last 4 min of the 6-minute testing period (*29*). The FST for each mouse was conducted individually.

### Further chronic unpredictable mild stress test

#### Treatments in chronic unpredictable mild stress test

CUMS test was carried out in order to determine the optimal mass fraction of eleutheroside with the best antidepressant effect. Another 60 mice were divided into six groups (*N*=10) randomly. There were normal control group, model control group, CH (40 mg/kg) group, and functional beverage (3.75, 7.50 and 15.00 mg/kg of eleutheroside) groups. Note that the functional beverage mass fractions were adjusted in CUMS test based on the results of the preliminary acute stress experiment. The functional beverage group (30.00 mg/kg) was removed and new dose group (3.75 mg/kg) was added. Mice in normal control group and model control group were fed with normal saline. All mice were fed once a day by oral gavage at 0.02 mL/g body mass for three weeks. The mice were not anesthetized before gavage.

Normal control group was not stimulated. Other mice were subjected to CUMS as described by Kaye *et al.* (*30*) with some modiﬁcations. Animals were subjected to stress paradigm randomly once a day over a period of three weeks. The order of stressors is shown in Table 1. After three weeks of stress, an open field test and a sucrose preference test were conducted for each mouse.

**Table 1:** The arrangement of random stress for animals

*t*/week				*t*/day			
1	2	3	4	5	6	7
1	F	E	T	O	W	S	C
2	W	O	C	S	E	T	F
3	C	E	W	F	T	S	O

F=food deprivation for 24 h, E=exposure to empty water bottles for 24 h, T=tail pinch (60 s), O=overnight illumination, W=exposure to wet caging (200 mL of water into the sawdust bedding) for 24 h, S=cold water swimming for 5 min at 4 °C, C=tilted cage at 45 degrees for 24 h

#### Open field test

A 40 cm high, 80 cm long and 80 cm wide case was prepared for this experiment. The case was separated into 25 equal areas (16 cm by 16 cm) by drawing black lines. Each mouse was placed at the center of the case at the beginning and allowed to move freely in this open field case. Within the 3-minute test, the time that the mouse stays in the center area, the number of times that the mouse moves across lines, the number of stand-up times and the number of stools were recorded (*31*).

#### Sucrose preference test

Sucrose preference test was employed herein to determine the anhedonia, which is one of the core symptoms of major depression in humans (*32*). After chronic stress experiment, each mouse was offered two bottles of water (one with water and the other one with water with 29.24 mM sucrose). This experiment began at 4:00 pm after CUMS and ended at 8:00 am of the second day. Water and sucrose intakes were calculated by weighing each bottle. Then, the sucrose preference was calculated according to the following equation:

*w*(sucrose preference)=*m*(sucrose)/[*m*(sucrose)+*m*(water)] /1/

where *m*(sucrose) is the mass of sucrose water intake in mice and *m*(water) is the mass of water intake in mice.

#### Determination of monoamine and antioxidation

Mice after CUMS were sacrificed by cervical dislocation on the ice pack and their brains were isolated and weighed. Brain homogenates were prepared manually from normal saline and mice brain tissues in a ratio of 9:1 (by mass). The supernates were stored in a -80 °C environment after centrifugation at 15 000×*g* and 4 °C for 10 min (H1650; Xiangyi Centrifuge Instruments, Changsha, Hunan, PR China). Levels of dopamine (DA), 5-hydroxytryptamine (5-HT) and norepinephrine (NE) were measured by enzyme-linked immunosorbent assay (ELISA) kits (H710, H104, H096; Jiancheng Bioengineering Institute, Nanjing, Jiangsu, PR China) using a microplate reader (Model 680; Bio-Rad Laboratories, Redmond, WA, USA). Superoxide dismutase (SOD) and malondialdehyde (MDA) were determined by WST-1 method and thiobarbituric acid (TBA) colorimetry by kits (A001-3 and A003-1; Jiancheng Bioengineering Institute) using the above-mentioned microplate reader.

### Statistical analysis

All the results are expressed as mean value±standard deviation (S.D.). The data were analyzed by one-way ANOVA test and Duncan’s test at p<0.05 and p<0.01 using the IBM SPSS Statistics v. 20.0 software (*33*).

## RESULTS AND DISCUSSION

### Effects of functional beverage on mouse behavior after preliminary acute stress experiment

The emotional despair is one of the core symptoms of depression and has causal relevance to committing suicide (*34*). TST is a desperate model induced by the inability to overcome abnormal postures and FST induces despair through the inability to escape the water environment (*35*, *36*). In addition, another symptom of depression is psychomotor retardation manifested as the reduction in locomotor activity in rodents, also known as immobility (*26*). Because the main measures of TST and FST are the reduction of locomotor activity and they contain a desperate environment, they are commonly used as rodent depression models (*27*).

**Fig. 1:**
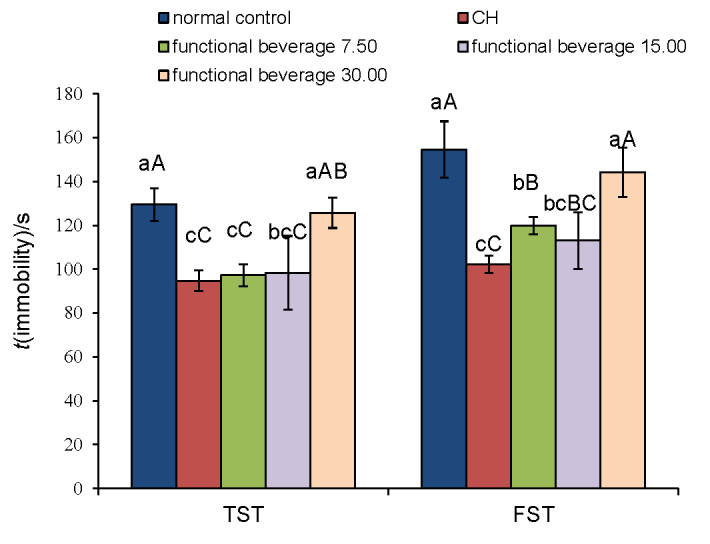
Effect of functional beverage on the immobility time of tail suspension test (TST) and forced swimming test (FST) in mice. Data are expressed as mean value±S.D. (*N*=10). Different superscripted lowercase letters in the same half region (TST or FST) denote significant differences according to Duncan’s test (p<0.05). Different superscripted capital letters in the same half region (TST or FST) denote highly significant differences according to Duncan’s test (p<0.01). CH=clomipramine hydrochloride. Functional beverages 7.50, 15.00 and 30.00 refer to the addition of eleutheroside in mg/kg

The effect of functional beverage on the immobility time of TST and FST is shown in Fig. 1. The immobility time in TST and FST was very significantly reduced in the dose groups of 7.50 and 15.00 mg/kg (p<0.01) as well as in the positive control CH group compared to the control group. The dose group of 30.00 mg/kg had no significant difference (p>0.05) from the control group, which suggested that the dose of 30 mg/kg does not have antidepressant effect. The dose group of 7.50 and 15.00 mg/kg in TST did not show significant difference (p>0.05) from the CH group, which suggested that these dose groups can reach the same antidepressant effect as CH group.

These two doses (7.50 and 15.00 mg/kg) were chosen to be the reference doses for follow-up experiments. Since the highest mass fraction (30.00 mg/kg) did not have antidepressant effect, it was necessary to explore the effect of lower mass fraction in order to obtain the most appropriate mass fractions of eleutheroside. Therefore, the dose group of 3.75 mg/kg was added in the follow-up experiments.

### Effects of functional beverage in mice after chronic unpredictable mild stress test

#### Effects on mouse behavior

The chronic unpredictable stress model was proposed by Katz *et al.* (*37*). The mice suffered a series of different stress stimuli including tail clip, cold-water swimming, and day-night reversal within 21 days, and these stimuli were randomly arranged. After the stimulation, the mice showed a series of emotional behavioral changes, such as reducing horizontal activity and the ability of exploration (*38*). Lu *et al.* (*39*) demonstrated that CUMS-induced depression-like behaviors are coupled with DAergic hyperfunction in the nucleus accumbens and serotonergic hypofunction in the hippocampus and prefrontal cortex.

The results of the open field test and the sucrose preference test are shown in Table 2. All measurements of model group were very significantly different from the control group (p<0.01), which proved the validity of this chronic mild stress model. The model control group showed significant differences from the CH group, the dose groups of 7.50 and 15.00 mg/kg in sucrose preference, the number of the lattice moved, the stand-up times, immobility time, and the number of stool grains (p<0.05), except the dose group of 3.75 mg/kg in the stand-up times.

**Table 2:** Effect of the functional beverage mass fraction, expressed as eleutheroside, on the open field test and the sucrose preference test in mice

Group	*w*(eleutheroside)/(mg/kg)	*w*(sucrose preference)/%	*N*(lattice moved)	Stand-up times	*t*(immobility)/s	*N*(stool grain)
Normal control		(68.2±6.2)^abA^	(91.0±5.2)^bcA^	(24.7±3.2)^abA^	(13.2±2.8)^cCD^	(1.4±1.0)^cB^
Model control		(43.0±4.4)^cB^	(56.7±3.9)^eC^	(10.4±3.2)^cB^	(34.9±8.9)^aA^	(396±1.1)^aA^
CH		(76.0±12.8)^aA^	(98.8±9.7)^abA^	(26.7±2.8)^aA^	(10.8±3.6)^cD^	(1.1±0.7)^cB^
Functional beverage	3.75	(48.5±8.2)^cB^	(69.7±8.6)^dB^	(12.6±2.8)^cB^	(22.9±4.8)^bB^	(2.7±01.0^bAB^
	7.50	(66.9±8.9)^abA^	(88.0±7.2)^cA^	(21.9±4.4)^bA^	(13.3±4.5)^cCD^	(1.3±1.6)^cB^
	15.00	(71.9±14.2)^abA^	(100.4±7.9)^aA^	(26.1±6.5)^abA^	(11.0±3.4)^cD^	(1.1±0.9)^cB^

Data are expressed as mean value±S.D. (*N*=10). Different superscripted lowercase letters in the same column denote significant differences according to Duncan’s test (p<0.05). Different superscripted capital letters in the same column denote highly significant differences according to Duncan’s test (p<0.01). CH=clomipramine hydrochloride

#### Contents of dopamine, norepinephrine and 5-hydroxytryptamine

After accidental finding that monoamine oxidase can inhibit iproniazid, monoamine hypothesis of depression was formulated, which stated that deficiency of monoamine neurotransmitters underlies clinical depression and depressive symptoms can be alleviated by increased monoamine (*40*-*42*). Currently, levels of monoamine such as NE, 5-HT and DA are often used as indicators in antidepressant research.

The effect of functional beverage on the contents of DA, NE and 5-HT in mice’s brains is summarized in Table 3. Compared to the normal control group, the contents of DA, NE and 5-HT in mice’s brains of the model control group decreased highly significantly (p<0.01), which denoted again that the mouse model of depression was established successfully. The contents of DA, NE and 5-HT of the dose groups of 7.50 and 15.00 mg/kg significantly increased in comparison with the model group (p<0.05). The dose group of 3.75 mg/kg did not show significant difference (p>0.05) from the model control group. These results were consistent with the effects on sucrose preference.

**Table 3:** Effect of the functional beverage mass fraction, expressed as eleutheroside, on the content of dopamine (DA), norepinephrine (NE) and 5-hydroxytryptamine (5-HT) in the brain of mice

Group	*w*(eleutheroside)/(mg/kg)		*γ*(DA)/(pg/mL)	*γ*(NE)/(pg/mL)	*γ*(5-HT)/(pg/mL)
Normal control		(22.3±2.2)^abAB^		(70.5±8.5)^bcdBC^	(95.6±15.7)^aA^
Model control		(16.7±2.1)^dC^		(56.0±5.1)^eD^	(71.4±2.9)^cC^
CH		(23.1±2.8)^aAB^		(86.1±9.6)^aA^	(98.0±6.3)^aA^
Functional beverage	3.75	(18.6±1.8)^cdBC^		(62.2±9.2)^deCD^	(80.2±9.0)^bcBC^
	7.50	(21.5±2.7)^abcABC^		(73.3±8.8)^bcABC^	(88.4±11.4)^abAB^
	15.00	(23.8±3.9)^aA^		(79.2±11.1)^abAB^	(99.0±10.3)^aA^

Data are expressed as mean value±S.D. (*N*=10). Different superscripted lowercase letters in the same column denote significant differences according to Duncan’s test (p<0.05). Different superscripted capital letters in the same column denote highly significant differences according to Duncan’s test (p<0.01). CH=clomipramine hydrochloride

#### Changes of superoxide dismutase activity and malondialdehyde content

Oxidative alterations are recognized as a critical route of brain damage in the pathophysiology of stress-induced psychiatric disorders (*43*). In stress disorders, oxidative stress triggers or exacerbates several routes of damage such as mitochondrial dysfunction, dysregulation of calcium homeostasis, disruption of energy pathways, damage to neuronal precursors, impairment of neurogenesis and induction of signaling events in apoptotic cell death (*44*). Oxidative stress is caused by an imbalance between the levels of free radical production and efficiency of the antioxidant enzyme system to neutralize and eliminate reactive oxygen species (ROS). Free-radical damage by ROS, such as the superoxide anion and hydrogen peroxide, is the primary source of oxidative stress (*45*). Two main antioxidant systems exist. The nonenzymatic system relies on molecules that can directly quench ROS and the enzymatic system is composed of specific enzymes that detoxify ROS. Among the latter, the SOD family is important in oxidative stress modulation (*46*). In addition, ROS levels are associated with lipid antioxidant defenses. The specific reduction in lipid-targeted antioxidant defenses may contribute to increased ROS levels and oxidative damage to lipid membranes (lipid peroxidation) including to polyunsaturated fatty acids. Lipid hydroperoxide chain reactions eventually cause the formation of reactive aldehydes, the end-product of lipid peroxidation, as indicated by increased levels of MDA (*47*).

Therefore, SOD activity and MDA content in the brains of mice were measured to reflect oxidative alterations. Whether SOD or MDA, all the functional beverage groups were significantly different compared with the model control group (p<0.05) (Table 4). The dose group of 15.00 mg/kg has the best effect in increasing SOD activity.

**Table 4:** Effect of functional beverage mass fraction, expressed as eleutheroside, on the content of superoxide dismutase (SOD) and malondialdehyde (MDA) in in protein of the mice brain

Group	*w*(eleutheroside)/(mg/kg)	Specific activity(SOD)/(U/mg)	*b*(MDA)/(mmol/g)
Normal control		(402.6±73.2)^aA^	(66.5±6.2)^dC^
Model control		(203.1±12.7)^dC^	(157.7±18.0)^aA^
CH		(402.5±47.2)^aA^	(94.4±11.8)^bB^
Functional beverage	3.75	(277.2±51.1)^bcBC^	(89.7±17.1)^bcB^
	7.50	(313.1±66.3)^bB^	(77.8±10.6)^cdBC^
	15.00	(417.1±58.7)^aA^	(86.5±10.5)^bcB^

Data were expressed as mean value±S.D. (*N*=10). Different superscripted lowercase letters in the same column denote significant differences according to Duncan’s test (p<0.05). Different superscripted capital letters in the same column denote highly significant differences according to Duncan’s test (p<0.01). CH=clomipramine hydrochloride.

## CONCLUSIONS

In conclusion, the treatment using functional beverage with 15.00 mg/kg eleutheroside had the best antidepressant effect, which significantly reduced the immobility time of mice in the tail suspension test and forced swimming test, and recovered sucrose preference degree. It also significantly improved the content of dopamine, norepinephrine, 5-hydroxytryptamine and the activity of SOD, and decreased the content of MDA in mice’s brains, which indicated that the improvement of monoamine neurotransmitter systems and antioxidation was a potential mechanism of antidepressant action. The results implied that the functional beverage made of eleutheroside, active peptides and menthol may be consumed by humans to achieve antidepressant effect.
